# Comparative Evaluation of Caries Excavation Efficacy of Gentle Hand Bur System and Spoon Excavator in Primary Molars: A Randomized Controlled Study

**DOI:** 10.7759/cureus.93681

**Published:** 2025-10-01

**Authors:** Anuja A Gaikwad, Poonacha K. S., Saylee Deshmukh, Riddhika Shah, Sushil Chavan, Mrunal Borawake

**Affiliations:** 1 Pediatric Dentistry, K. M. Shah Dental College and Hospital, Vadodara, IND

**Keywords:** caries removal efficacy, dental caries, hand excavation, pain perception, pediatric dental care

## Abstract

Introduction: Dental caries is a major global health concern, especially among children in low-income regions, where untreated cases can lead to pain, infection, and loss of function. To reduce treatment-related anxiety and discomfort, minimally invasive methods like atraumatic restorative treatment (ART), chemo-mechanical removal, and manual tools such as spoon excavators (SEs) have been developed. These techniques focus on preserving healthy dentin and avoiding the noise and vibration of the airotor handpiece. The Gentle Hand Bur System (GHBS) builds on this approach, offering a quiet, vibration-free alternative aimed at improving comfort and reducing fear in pediatric patients.

Aim: This study aims to compare and evaluate caries excavation efficacy and pain perception while using the GHBS and the SE in primary molars.

Methodology: The study was designed as a randomized controlled trial and conducted among children aged four to nine years who required restorative treatment in at least two primary molars. The final sample size consisted of 60 molars, i.e., 30 in each group. Caries removal was done utilizing SE or GHBS, according to the allocated group. The caries removal efficacy was evaluated using a caries-detecting dye, and postoperative pain was assessed using a four-point pain intensity scale.

Results: On comparison of the mean values of the SE caries removal score (0.27) and GHBS caries removal score (0.40), the mean values were statistically not significant (p=0.293). Additionally, when the values of the SE pain perception score (1) and GHBS pain perception score (1) were compared, the mean values were statistically not significant (p=1.0).

Conclusion: When it comes to primary molar caries removal effectiveness and pain perception, the GHBS is on par with the traditional SE.

## Introduction

Dental caries is a serious health issue. Untreated carious deciduous teeth are the 10th most prevalent disorder, affecting millions of children globally. People from low-income countries and those from disadvantaged backgrounds are more likely to have dental cavities than people from high-income countries [1]. A child's quality of life can also be adversely affected by dental caries, leading to chewing difficulties, tooth loss, weight loss, behavioral problems, poor academic performance, and delayed cognitive development [2].

In the late 1800s, the rules for operative dentistry were coined by Dr. G. V. Black, the father of modern dentistry. Among the various principles given, the principle of "prevention of extension" is very important. The core idea behind this principle is to stop tooth decay without removing more of the healthy tooth structure than necessary. The "Minimal Invasive Dentistry" approach involves carefully removing only the decayed and infected parts of the tooth, while preserving as much of the potentially healthy tooth structure as possible, especially the inner layer of dentin, which can rebuild itself [3].

The use of an airotor allows for rapid and effective removal of carious tissue; however, it may also lead to the unnecessary elimination of sound dentin or affected dentin that possesses the potential for remineralization [4-6]. The sound produced by the airotor, the water spray used as coolant, and the simultaneous use of a suction apparatus may induce different types of stress in pediatric patients. Caries excavation using a spoon excavator (SE) is the most efficient method for the accurate removal of infected or soft dentin, especially when cutting is required adjacent to important anatomical structures [7]. The hand excavator can remove softened tissue with more sensitive tactile feedback than a bur, and this method is the more self-limiting method for caries excavation [8].

To facilitate minimally invasive tooth preparation and to simplify the caries removal procedure, a new carbide hand bur system named "Gentle Hand Bur System" (GHBS) was introduced by Gerry Beauchemin in 2022, which requires no handpiece or airotor for its operation. This bur system has two variants. The first variant includes a set of four Gentle Hand Bur (GHB) premium handles, while the second variant consists of only a single handle. The "P" handle, also known as the Premium-Lock handle, features a standard, round, threaded aluminum design incorporating a micro-chuck assembly along with a perforated pin-locking mechanism. According to the manufacturer, the GHBS offers several benefits, including smooth operation and eliminating the need for a handpiece or an airotor. As a result, it produces no vibration, noise, or pain, which contributes to reduced dental anxiety, particularly in pediatric patients [9].

Thus, this study aimed to compare the efficacy of caries excavation and pain perception when using the SE and the GHBS for caries excavation. According to the null hypothesis, there would be no significant difference in caries excavation efficacy and pain perception between the GHBS and the SE in primary molars.

## Materials and methods

Study design and setting

The proposed study was conducted as a single-blinded randomized controlled trial. Children between the ages of four and nine years old were selected from the Department of Pediatric and Preventive Dentistry, K. M. Shah Dental College and Hospital. The detailed information regarding the procedure was explained to the children and their parents; the information sheet was given to the parents. Written consent was obtained from the participant's parent, and verbal assent was taken from the child. Prior permission was taken from the Institutional Ethical Committee (approval no. SVIEC|ON|DENT|BVPG22|May|23|55). The trial was also registered with the Clinical Trials Registry-India (CTRI) prior to the recruitment of participants (CTRI/2024/02/062368).

Sample size

With 5% alpha error, 95% power of the study, and a clinically significant difference of 0.8 units, the required sample size was 50 primary molars. Considering a 20% dropout rate, the final sample size was 60 primary molars.

Eligibility

Inclusion Criteria

The study included children aged four to nine years old, children who had occlusal carious lesions in multiples of two teeth on the maxillary and/or mandibular first or second primary molars, those with teeth classified under Mount and Hume caries classification number 1.2, children whose parents provided written consent for the study, and children with Frankel's behavior rating scale 3 and 4.

Exclusion Criteria

The study excluded children with pulpal, periodontal, and soft tissue pathology affecting the affected teeth, nonvital teeth, and those with any systemic disease or congenital abnormality.

Methodology

After considering all the inclusion and exclusion criteria, a total of 60 molars with occlusal caries were selected for the study. All the participants were thoroughly examined by the principal investigator on their first visit. The proper training of the technique used for caries removal with the GHBS was provided by the principal investigator. The selected teeth were randomly allocated to one of the two treatment groups using a computer-generated system by the principal investigator. The involved tooth was isolated using a rubber dam.

Group One (Control Group)

**Figure 1:**
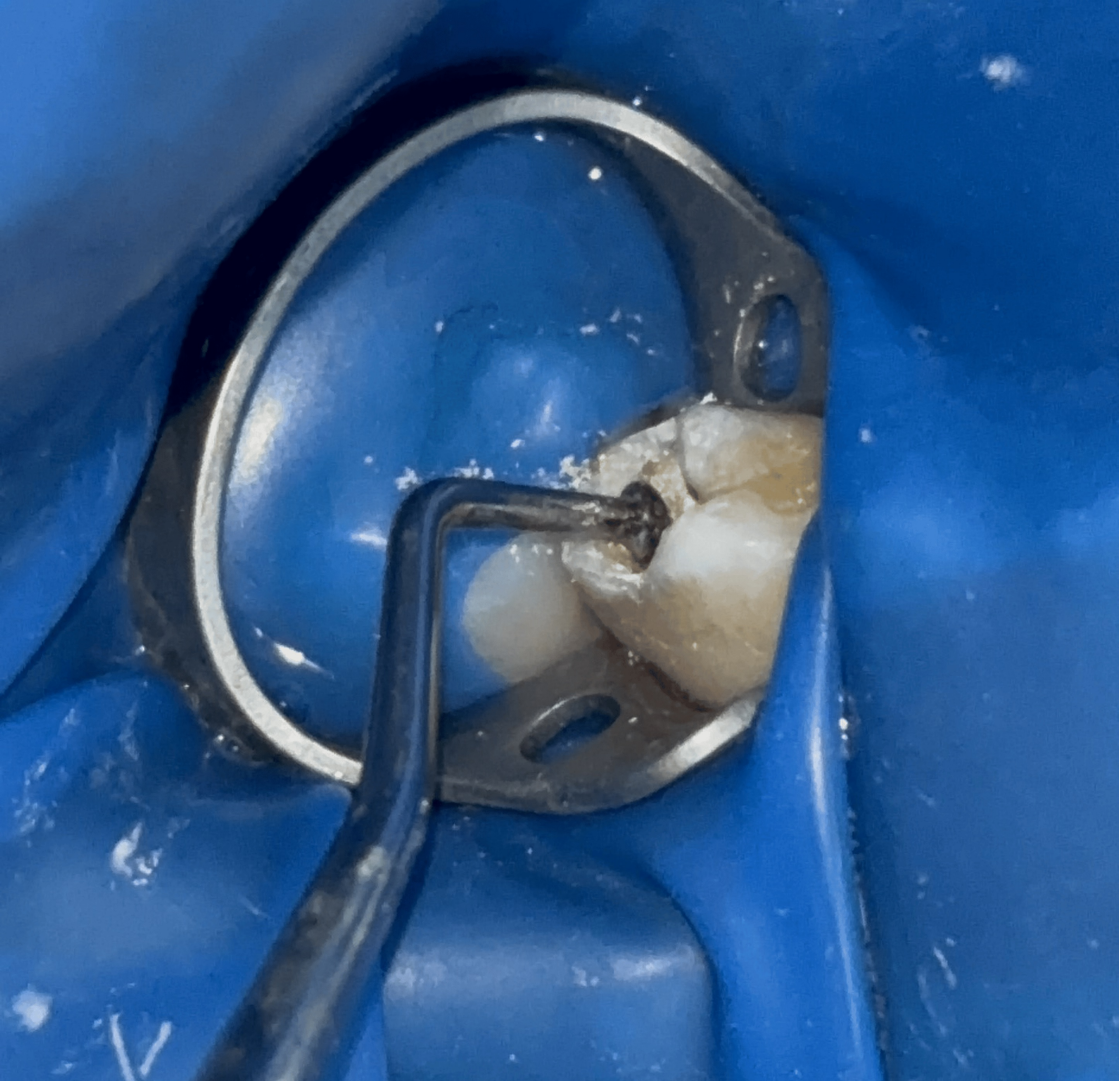
Caries excavation using the spoon excavator

The caries excavation was done using a sterile SE. The motions used were the circular scooping movements of the SE around the long axis of the instrument. During the excavation, dentin hardness was assessed, and caries removal was completed when hard dentin was encountered using a Shepherd hook explorer. Depending on the size of the carious lesion, different-sized SEs were used (Figure 1).

Group Two (Experimental Group)

**Figure 2:**
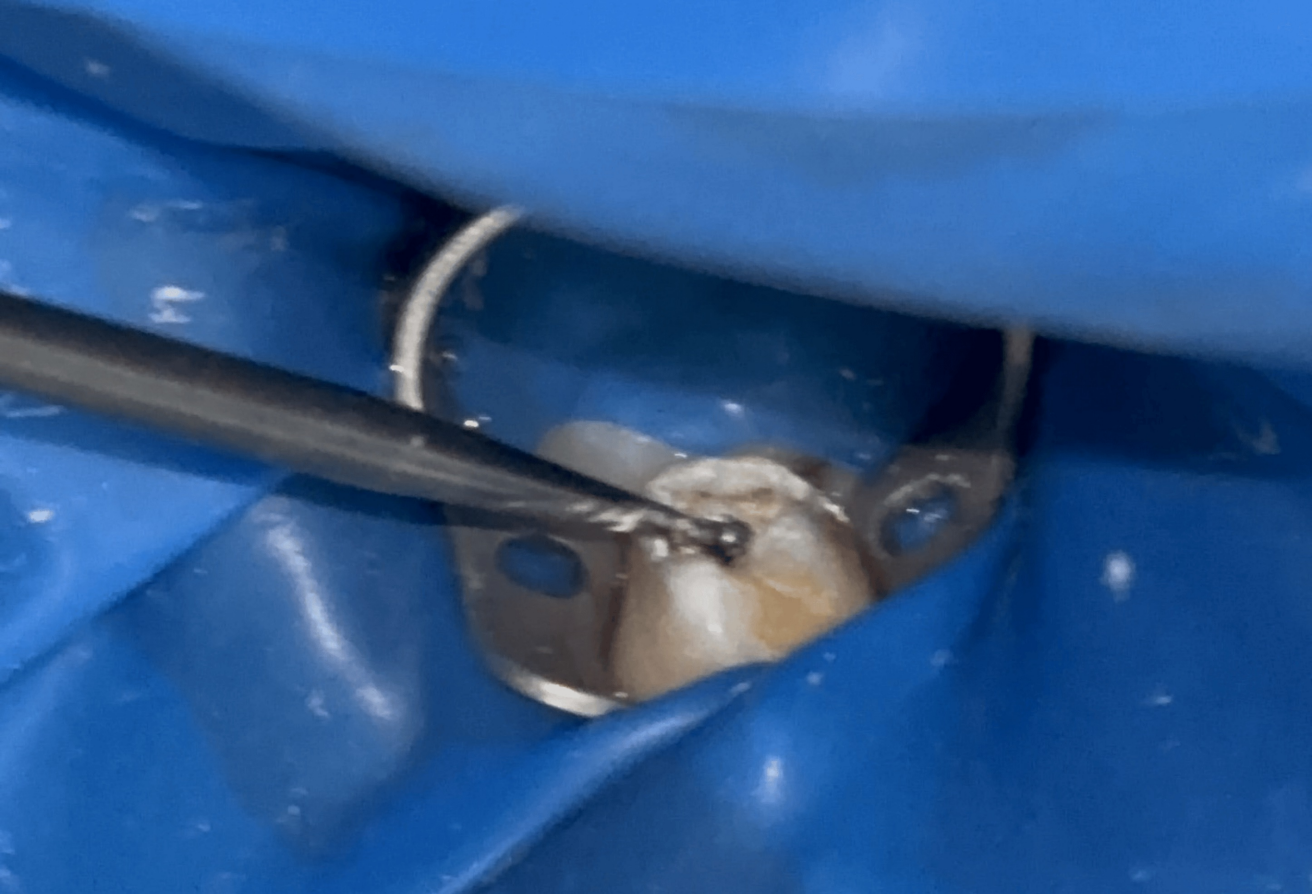
Caries excavation using the Gentle Hand Bur System

The caries were excavated using the GHBS, utilizing rotational motion around the long axis of the instrument's handle. The rotatory movements were performed, starting from the center of the lesion to the periphery, from the occlusal aspect, without the use of water coolant. The caries removal was terminated after hard dentin was detected using a shepherd hook explorer (Figure 2).

Assessment of clinical parameters

The pain threshold was evaluated during caries removal using a four-point pain intensity scale, as assessed by the principal investigator, and categorized as follows: 0 (no pain), 1 (slight pain), 2 (moderate pain), and 3 (severe pain) [10]. The efficacy of caries removal was verified using caries-detecting dye as recommended by the manufacturer. One drop of caries-detecting dye (Caries Detector, Waldent, New Delhi, India) was placed on a sponge pledget and applied to the cavity for 10 seconds. Then, it was rinsed with water. "The cavity was checked for remaining caries using an explorer. The completeness of caries removal was judged by the clinical criteria that a sharp explorer should not stick in the dentine and not give a 'tug-back' sensation" [11]. Caries removal was rated according to scoring criteria given by Munshi et al. as 0 = Caries removed completely; 1 = Caries present in the base of the cavity; 2 = Caries present in the base and/or one wall; 3 = Caries present in the base and/or two walls; 4 = Caries present in the base and/or more than two walls; and 5 = Caries present in the base, walls, and margins of the cavity [12].

The teeth were restored with either Glass Ionomer Cement (GIC) or composite restoration (which was mixed according to the manufacturer's instructions) as per the choice of the parents in both the treatment groups. After the restoration of the cavity, high points were checked using articulating paper. In the event of pulpal exposure, the principal investigator carried out the appropriate treatment, and the patient was not considered a study subject.

Statistical analysis

The data collected were entered into a Microsoft Excel spreadsheet (Microsoft Corporation, Redmond, Washington). Descriptive statistical tests were computed using Excel statistical operations. Inferential statistics was done using IBM SPSS Statistics for Windows, Version 23 (Released 2015; IBM Corp., Armonk, New York). Descriptive statistical parameters used were means and standard deviations. The data were analyzed using the paired t-test and the Wilcoxon signed-rank test. The level of significance was set at p < 0.05, and any value less than or equal to 0.05 was considered statistically significant. A paired t-test was used to find the significance of study parameters on a continuous scale between two groups.

## Results

Table 1 depicts the mean GHBS caries removal score (0.40), which is numerically higher than the mean SE caries removal score (0.27). This suggests that, on average, the GHBS scoring system tended to assign slightly higher scores, indicating potentially less complete caries removal according to criteria suggested by Munshi et al. [12]. The mean difference of -0.13 (GHBS - SE) further confirms that the GHBS scores were, on average, 0.13 units higher than the SE scores for the same molars. Based on the results of the paired t-test, there is no statistically significant difference (p = 0.293) in the mean caries removal scores obtained using the SE and GHBS.

**Table 1:** Comparison of the Spoon Excavator (SE) and Gentle Hand Bur System (GHBS) caries removal scores using a paired t-test A p-value of less than or equal to 0.05 is considered statistically significant.

Method	N (%)	Mean ± SD	Mean Difference ± SD	T	p-value
SE	30 (50%)	0.27±0.45	-0.13±0.68	-1.07	0.293
GHBS	30 (50%)	0.40±0.62			

When the caries removal score was plotted in box and whisker format, it was evident that both groups showed a similar lower quartile, indicating that 25% of the molars had a score of 0. This suggests that at least a quarter of the samples were assessed as having complete caries removal by both methods. The most noticeable difference was in the higher upper whisker for the GHBS scores (around 2) compared to the SE scores (around 1). This indicates that the GHBS recorded higher maximum scores for caries removal compared to the SE system on the same set of molars.

Table 2 depicts a mean pain perception score of 1 for both groups, which suggests that, on average, the reported pain level falls into the slight pain category. The SD of 0.37 for the SE score suggests that most individual scores are likely to be around 1, with some possibly falling into 0 (no pain) or 2 (moderate pain). It is less likely to see scores of 3 (severe pain) given this mean and standard deviation. Similarly, the SD of 0.46 for the GHBS score suggests that individual scores are also likely centered around 1. The p-value (1) indicates that there is no statistically significant difference in pain perception scores between the two groups.

**Table 2:** Comparison of the Spoon Excavator (SE) and Gentle Hand Bur System (GHBS) pain perception scores using a paired t-test A p-value of less than or equal to 0.05 is considered statistically significant.

Method	N (%)	Mean ± SD	Mean Difference ± SD	t	p-value
SE	30 (50%)	1±0.37	0±0.46	0.00	1
GHBS	30 (50%)	1±0.46			

## Discussion

Within dental practice, the importance of conserving tooth structure, coupled with a patient-centered approach, is increasingly recognized. By using minimally invasive restorative dentistry treatments, the sound tissue can be preserved and shielded from harm, ensuring the natural tooth has the longest possible lifespan. Removing diseased dentin without compromising healthy dentin that can re-mineralize is the ultimate goal of restorative dentistry [13,14].

One of the primary goals of dental research has been to develop new caries excavation techniques that improve and replace conventional approaches [4]. Various alternative techniques, such as air abrasion, laser, and chemo-mechanical caries excavation, have been demonstrated to be more or less successful in resolving these issues. When compared to preparation, cost, and space, the majority of these techniques take almost as much time [14].

It is well recognized that anxiety and fear undermine oral health and make people less receptive to dental treatment. Traditional drilling methods cause discomfort, particularly for young patients. Additionally, a drill eliminates both the infected and affected dentin, resulting in significant destruction of healthy tooth structure [6].

Dental professionals often prefer hand excavation when working with uncooperative children or when performing stepwise excavation. In comparison to the bur, hand excavation offers superior sensory control and less strain without producing high temperatures [14]. Numerous studies have demonstrated that using an SE to remove dental cavities is less painful than using an airotor [15,16]. Celiberti et al. demonstrated that, in addition to having a decent excavation time and the capacity to remove caries effectively, a hand excavator appeared to be the most suitable technique for carious dentine removal in primary teeth [15]. The caries excavation methods employed in this study are types of manual excavation methods, namely, the SE and the GHBS. Both methods adhere to the principles of minimally invasive dentistry.

SE is a traditional method of hand excavation, while the GHBS is a relatively new method introduced for caries excavation. There were no studies that compared the caries removal efficacy and difference in pain perception between these two groups; hence, the current study was conceptualized.

The caries removal efficacy was checked using a caries-detecting dye. Hosoya et al. examined the effectiveness of caries-detecting dyes based on propylene glycol on both primary and permanent teeth. They noted the high diffusion capability of propylene glycol, attributed to its lower molecular weight and surface tension, which could result in deeper dye infiltration into intact dentin. Hence, it was proposed that employing polypropylene glycol, which possesses a greater molecular weight, may hinder the penetration of propylene glycol into sound dentin [18].

Pain is widely recognized as the fifth vital sign and remains a key reason why individuals seek medical care [18]. Accurate and reliable measures of pain are essential to reflect the actual intensity experienced. However, children's limited verbal abilities can hinder effective pain communication. As a result, multiple pain assessment tools have been developed to support pediatric patients in self-reporting their pain [19]. The four-point pain intensity scale is among several facial expression-based tools that have been validated for pain assessment across diverse pediatric settings [20]. It has been shown that most children aged three years and older can utilize this scale both effectively and accurately, with its validity and reliability confirmed in studies involving children aged three to 18 years across different environments and populations [21]. Therefore, the four-point pain intensity scale was employed in our study to evaluate pain perception.

This study aimed to compare the caries removal efficacy and pain perception between the GHBS and the conventional SE in primary molars. Our findings revealed no statistically significant difference in caries removal efficacy, as assessed by the Munshi et al. scoring criteria using a caries-detecting dye, between the two methods (p = 0.293 in the paired t-test). Similarly, there was no statistically significant difference in the reported pain perception between the GHBS and the SE (p = 1.00 in the paired t-test), with the majority of children in both groups reporting "slight pain." Thus, the null hypothesis, stating that there would be no difference between caries excavation efficacy with the GHBS and SE in primary molars, could not be rejected based on our findings.

The comparable caries removal efficacy between the GHBS and the SE is an interesting finding. While the GHBS is marketed as a less invasive and potentially more comfortable alternative to traditional rotary instruments, our results suggest that it is equally effective as the conventional SE in removing caries, as judged by the residual caries score after excavation.

The observation that the mean caries removal score was numerically slightly higher for the GHBS group (indicating potentially more residual caries) warrants further consideration. Although this difference was not statistically significant, the wider interquartile range (IQR) observed in the GHBS group suggests a greater variability in the amount of residual caries left behind by this method. This could imply that the effectiveness of the GHBS might be more operator-dependent or influenced by the characteristics of the carious lesion.

Study limitations

The study had several limitations, including a small sample size and the subjective nature of caries detection using dye and the Ericson scale. It lacked advanced objective methods, such as Quantitative Light-Induced Fluorescence (QLF) and Micro-Computed Tomography (micro-CT), and faced limited accessibility of the GHBS in the maxillary posterior region.

## Conclusions

In conclusion, our study suggests that the GHBS is comparable to the conventional SE, with no statistically significant difference observed in terms of caries removal efficacy and pain perception in primary molars. While the GHBS may offer advantages in terms of avoiding the use of airotors, further research with a larger sample size and objective outcome measures is needed.
